# Tissue-Resident Macrophages: Linking Physiology and Inflammation in Infection

**DOI:** 10.1007/s12016-026-09195-x

**Published:** 2026-09-11

**Authors:** Marcelo Eduardo Cardozo, Tatyane Martins Cirilo, Isabela de Brito Duval, Ana Laura Grossi de Oliveira, Luisa Mourão Dias Magalhães, Lilian Lacerda Bueno, Eric Dumonteil, Ricardo Toshio Fujiwara

**Affiliations:** 1 Laboratory of Immunobiology and Parasite Control, Institute of Biological Sciences, Universidade Federal de Minas Gerais, Antônio Carlos Avenue, Pampulha Campus: Building E4/Room 173, Belo Horizonte, 6627 Brazil; 2 Post-graduation Program in Health Sciences: Infectious Diseases and Tropical Medicine, Faculdade de Medicina, Universidade Federal de Minas Gerais, Belo Horizonte, Brazil; 3 Laboratory of Interactions in ImmunoParasitology, Institute of Biological Sciences, Institute of Biological Sciences, Universidade Federal de Minas Gerais, Belo Horizonte, Brazil; 4 National Institute of Science and Technology for Research in Mucosa and Skin (INCT - Mucosa e Pele), Universidade Federal de Minas Gerais, Belo Horizonte, Brazil; 5 Department of Tropical Medicine and Infectious Disease, Celia Scott Weatherhead School of Public Health and Tropical Medicine, Infectious Disease Research Center, Vector-Borne, Tulane University, New Orleans, LA USA

**Keywords:** Host-pathogen interactions, Organ physiology, Infection immunology, Macrophage ontogeny, Neuroimmune interaction, Precision immunotherapy

## Abstract

Macrophages are found in every tissue in the body, and their importance goes far beyond clearing pathogens. Developmental studies have shown that many macrophage populations originate during embryogenesis, persist through self-renewal, and adopt transcriptional and functional programs defined by their tissue environment. These tissue-resident macrophages (TRMs) are embedded within organ physiology, where they regulate, for instance, cardiac conduction, sculpt synaptic circuits, recycle iron, maintain epithelial barriers, and coordinate repair. During infection, TRMs connect to neural, metabolic, and structural circuits; therefore, inflammatory activation can alter organ function long after pathogen clearance. We propose that TRMs function as physiological integrators of immunity, translating local environmental cues into immune responses constrained by the demands of their tissue. This review examines macrophage populations across interconnected physiological systems, including the heart–brain axis, the spleen–liver network, and barrier tissues. In these settings, developmental origin, niche-derived signals, and metabolic programming determine how macrophages respond to infection and whether they preserve or disrupt tissue homeostasis. Additionally, we discuss how loss of resident identity during inflammatory stress contributes to chronic pathology and post-infectious sequelae and how emerging therapeutic strategies aim to restore tissue-specific macrophage programs. By framing immunity within organ physiology, we offer a conceptual model for understanding how infection reshapes tissue function through resident macrophages, as well as how targeting these cells could prevent long-term damage.

**Graphical Abstract:**

**Figure:**
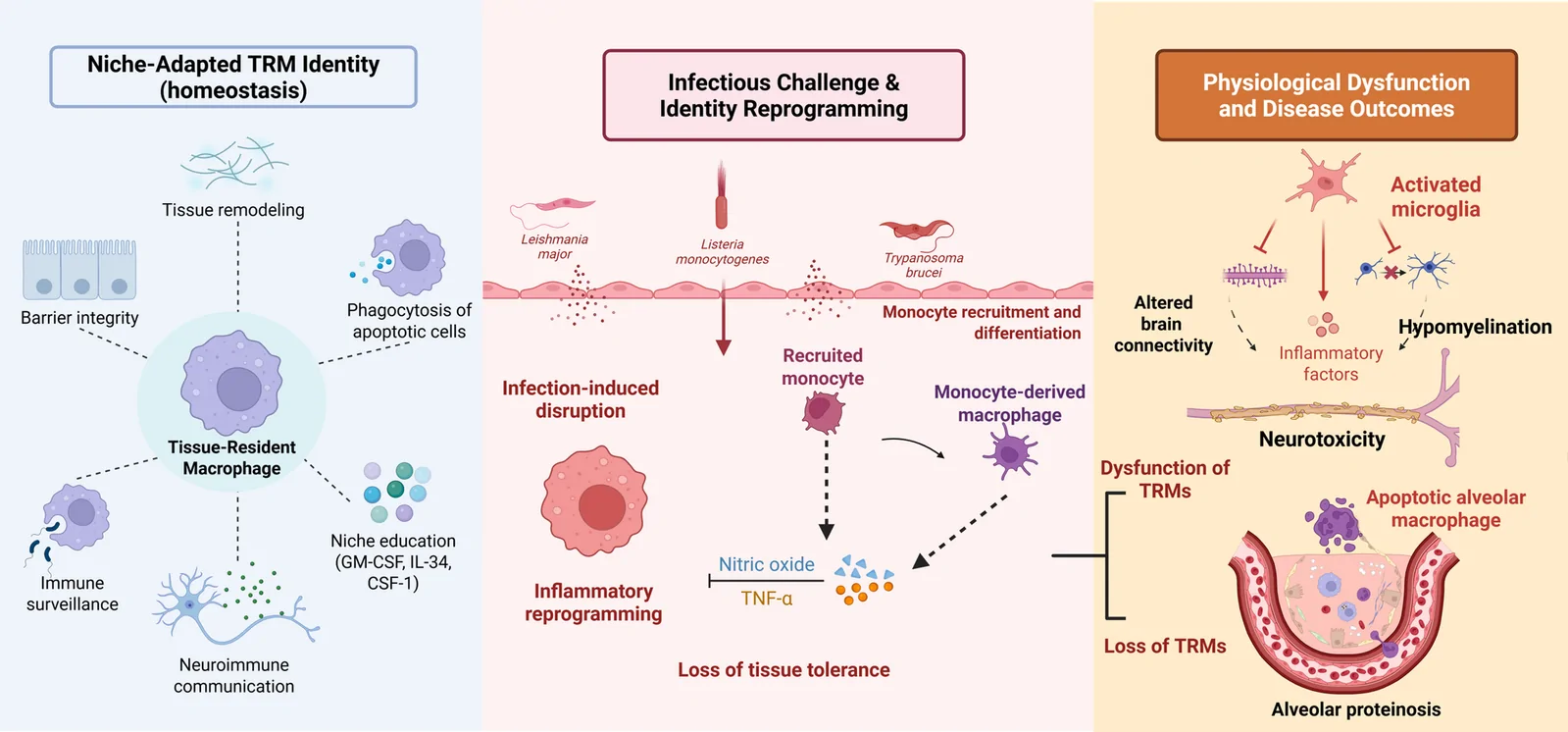

## Introduction

Macrophages populate every tissue; however, no two populations occupy identical niches or perform redundant functions. Previously viewed as primarily terminally differentiated descendants of circulating monocytes, macrophages are now recognized as a heterogeneous lineage whose identity emerges from the convergence of developmental origin, tissue residence, and inflammatory challenge [1–3]. Subsequent work emphasized that macrophages acquire distinct functional identities within different organs and contribute not only to host defense but also to tissue development, remodeling, and homeostasis [4].

**Fig. 1:**
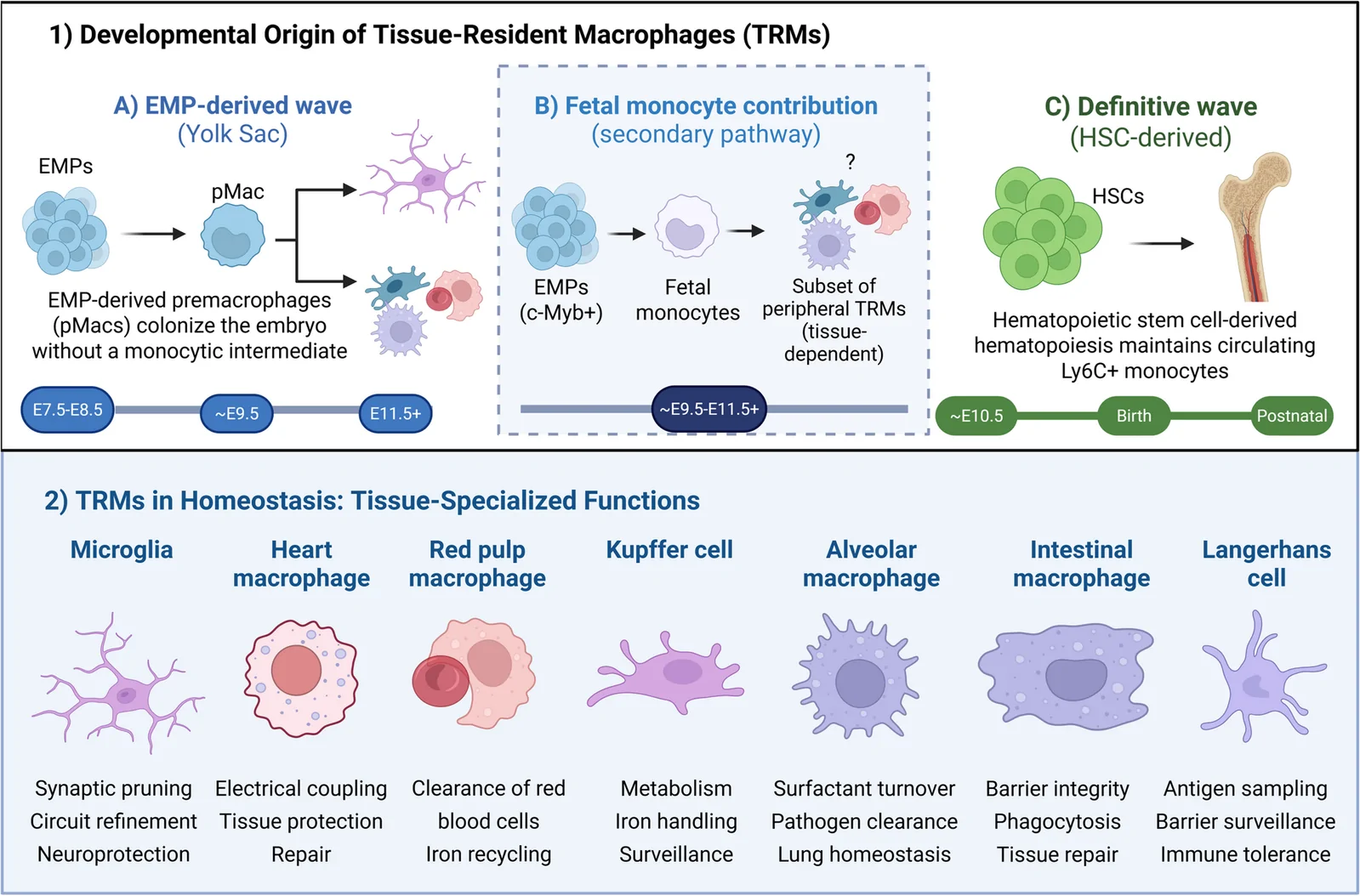
Developmental origin and tissue specialization of tissue-resident macrophages (TRMs). Erythro-myeloid progenitors (EMPs) emerge in the yolk sac and give rise to premacrophages (pMacs), which colonize the entire embryo without a monocytic intermediate and establish both microglia within the central nervous system and the founder populations of peripheral TRM compartments. A subset of c-Myb⁺ EMPs additionally generates fetal monocytes in the fetal liver, which contribute to a subset of peripheral TRM populations in a tissue-dependent manner. Later, hematopoietic stem cell (HSC)-derived definitive hematopoiesis, arising within the aorta–gonad–mesonephros (AGM) region, maintains circulating Ly6C+ monocytes that can be recruited during inflammation and tissue injury. Following tissue colonization, TRMs undergo niche-dependent education and acquire specialized programs that support tissue-specific physiological functions, including neural homeostasis, electrical coupling, iron recycling, metabolic regulation, surfactant turnover, barrier maintenance, and immune surveillance

Lineage-tracing studies in mice indicate that tissue-resident macrophage (TRM) populations are established during embryogenesis through successive and partially overlapping waves of hematopoiesis (Fig. 1) [5, 6]. Erythro-myeloid progenitors (EMPs) emerge in the yolk sac beginning at approximately embryonic day (E) 7.5–8.5 [7, 8]. EMPs give rise to premacrophages (pMacs), a CX3CR1-expressing intermediate that is developmentally distinct from monocytes, which simultaneously colonize the whole embryo from approximately E9.5 onward without passing through a monocytic intermediate [7, 9]. pMacs that colonize peripheral organs establish the founder populations of multiple peripheral TRM compartments. Depending on the tissue, these founder populations may subsequently be supplemented by additional embryonic precursors, including fetal monocytes, during development [6, 9, 10]. A subset of EMPs seeding the fetal liver upregulates c-Myb and generates fetal monocytes, which contribute to the establishment of selected peripheral TRM populations in a tissue-dependent manner [6, 9–11].

Later, definitive hematopoiesis arises from HSCs generated in the aorta–gonad–mesonephros (AGM) region, which seed the fetal liver and subsequently the bone marrow, establishing lifelong hematopoietic output and the circulating monocyte pools that replenish or supplement tissue macrophage compartments postnatally [12–14]. Taken together, these developmental pathways establish the embryonic foundation of tissue-resident macrophage biology, with the relative contribution of pMacs, fetal monocytes, and later definitive hematopoiesis varying substantially across tissues and developmental stages.

The relative contribution of each developmental wave to adult TRM populations varies across tissues and continues to be refined by emerging lineage-tracing, barcoding, and single-cell approaches [9–11]. Multiple studies support a contribution of EMP-derived fetal monocytes to peripheral TRM populations [6, 9, 13]. More recent clonal barcoding strategies have raised important questions regarding the relative contribution of fetal monocytes; therefore, the precise sequence and relative contribution of embryonic intermediates, including pMacs and fetal monocytes, remain an area of active investigation and are likely to differ among tissues and developmental stages [10]. First, once fetal monocytes contribute to the establishment of TRMs, the fetal compartment largely disappears, and the absence of a shared barcode at later stages may reflect this transience. Second, clonal barcoding can establish shared ancestry between EMPs and TRMs but cannot resolve whether fetal monocytes were the immediate precursor or whether tissue colonization occurred through an earlier EMP-derived macrophage intermediate. Third, incomplete temporal labeling during the induction window may systematically underestimate contributions from fetal monocytes generated outside that period. Furthermore, constitutive lineage-tracing models identify cells that activate a given promoter but do not directly establish developmental continuity between successive intermediates. Consequently, they cannot formally distinguish a direct pMac-to-TRM pathway from developmental trajectories involving additional embryonic intermediates that activate the same genetic locus [10, 11].

Moreover, tissue growth continues well beyond birth in humans, and the expansion of tissue-resident macrophage networks may involve circulating precursor contributions that differ from murine dynamics. Consistent with this, fate mapping using Ms4a3-Cre, which primarily labels definitive HSC-derived monocytes, demonstrates minimal contribution of this lineage to most tissue macrophage populations at birth, whereas pMac-specific fate-mapping (e.g., Tnfrs11a-Cre) labels the vast majority of the same populations, supporting pMacs as the principal EMP-derived intermediate for most TRM compartments [10, 11, 15].

Following tissue colonization, resident macrophages undergo extensive niche-dependent education, acquiring tissue-specific transcriptional, epigenetic, and functional programs that support local physiological demands [15, 16]. In parallel, circulating monocytes comprise functionally distinct subsets. Non-classical (Ly6C^low^) monocytes continuously patrol the vasculature under steady-state conditions, surveying endothelial integrity and responding to vascular pathogens [17, 18]. In contrast, classical monocytes (Ly6C^high^) are rapidly recruited to tissues during inflammation. Upon tissue entry, these cells can differentiate into macrophages under the influence of local microenvironmental cues [19, 20]. As a result, most organs contain two broad macrophage compartments: long-lived resident populations established during development and recruited macrophages generated from circulating monocytes in response to inflammatory stimuli [21, 22]. Although this didactic framework is conceptually useful, the relative contribution of each compartment varies considerably across tissues, inflammatory conditions, and species, generating intermediate populations with overlapping phenotypic and functional characteristics.

Embryonically derived TRMs are deeply integrated into organ physiology. Beyond their roles in immune defense, these cells contribute to apoptotic cell clearance, extracellular matrix remodeling, and the maintenance of local stem cell niches [23, 24]. In addition, TRMs perform highly specialized functions tailored to the physiological needs of each tissue (Fig. 1). For example, microglia regulate synaptic pruning in the central nervous system, Kupffer cells participate in systemic iron recycling in the liver, and cardiac macrophages contribute to electrical conduction within the heart [25–27]. These activities are indispensable for maintaining tissue homeostasis.

Understanding how such functional specialization arises, and why it is vulnerable to disruption during infection, requires appreciating interconnected principles that operate across all tissue macrophage populations. The first is developmental imprinting: early colonization by embryonic precursors establishes a baseline transcriptional landscape that is not readily accessible to acutely recruited adult monocyte-derived cells. While embryonically established chromatin programs confer a degree of resilience to inflammatory reprogramming, increasing evidence indicates that prolonged niche residence can drive substantial transcriptional and epigenetic convergence of monocyte-derived macrophages toward resident-like identities, although the extent of this convergence is tissue-dependent and may not fully recapitulate programs established by embryonic precursors [28, 29].

The second is niche education: local environmental signals continuously reinforce and maintain these transcriptional programs throughout life. GM-CSF secreted by alveolar type II epithelial cells drives PPAR-γ expression in lung macrophages [30], TGF-β and IL-34 from keratinocytes sustain Langerhans cell identity in the epidermis [31], heme released during erythrophagocytosis induces SPI-C expression in splenic red pulp macrophages [32], and CSF-1 from enteric neurons maintains muscularis macrophage survival in the gut wall [33]. Together, these niche-derived signals create an epigenetically stabilized identity in which H3K4me3 marks at homeostatic gene loci and H3K27me3 marks at inflammatory gene loci generate a chromatin architecture that resists acute reprogramming [34–36]. Loss of this stabilization during infection, through cytokine-driven displacement of identity transcription factors such as SALL1 in microglia or PPAR-γ in alveolar macrophages, promotes a transition toward an inflammatory activation state characterized by partial loss of tissue-resident identity and acquisition of inflammatory transcriptional programs [37, 38].

The third principle is ontogeny-dependent replacement susceptibility: tissues differ markedly in how readily their resident macrophage pool is replenished by monocytes following depletion, and the kinetics and transcriptional fidelity of this replacement depend on both the tissue niche and the developmental history of the original population [39–41]. These principles operate in concert to determine how macrophages become specialized physiological regulators within their respective organs, and how infection can durably alter organ function by disrupting this equilibrium.

In this review, we examine tissue-resident macrophages as organ-adapted immune cells whose functions emerge from the intersection of developmental origin, tissue niche, and infectious challenge. We organize our discussion around three interconnected physiological axes (Fig. 1). The first is the heart-brain neuroimmune axis, in which sympathetic nervous system activation links cardiac stress to systemic immune responses. The second is the liver–spleen metabolic immunity axis, where iron metabolism intersects with blood filtration and systemic pathogen control. The third encompasses barrier tissues, including the intestine, lung, and skin, where constant microbial exposure requires a finely tuned balance between immune tolerance and antimicrobial defense. These concepts provide a unifying framework for understanding tissue-resident macrophage biology across organs. Throughout this review, we highlight how developmental origin shapes the ability of macrophages to acquire and maintain tissue-specific physiological functions, how local niches continuously reinforce these programs, and how infection can disrupt this equilibrium through depletion, replacement, or inflammatory reprogramming of resident populations.

## Search Strategy and Selection Criteria

References for this Review were identified through searches of PubMed, Scopus, and Google Scholar for articles published up to 2026. The search was conducted using combinations of the following terms: “tissue-resident macrophages”, “macrophage ontogeny”, “neuroimmune interaction”, “heart-brain axis”, “iron metabolism”, “nutritional immunity”, “barrier tissues”, “alveolar macrophages”, “Kupffer cells”, “splenic macrophages” and “infection”. These terms were further combined with specific infection-related keywords, including “*Trypanosoma cruzi*”, “malaria”, “*Listeria monocytogenes*”, “influenza”, “helminth infection”, and “sepsis”. Additional relevant articles were identified through the authors’ personal files and manual searches of reference lists in key papers. Selection was prioritized for studies that utilized lineage tracing, single-cell transcriptomics, or specific genetic depletion models to distinguish between resident and recruited populations. We included articles published in English. Meeting abstracts and unpublished reports were excluded.

## The Heart-Brain Axis: Neuroimmune Integration

**Fig. 2:**
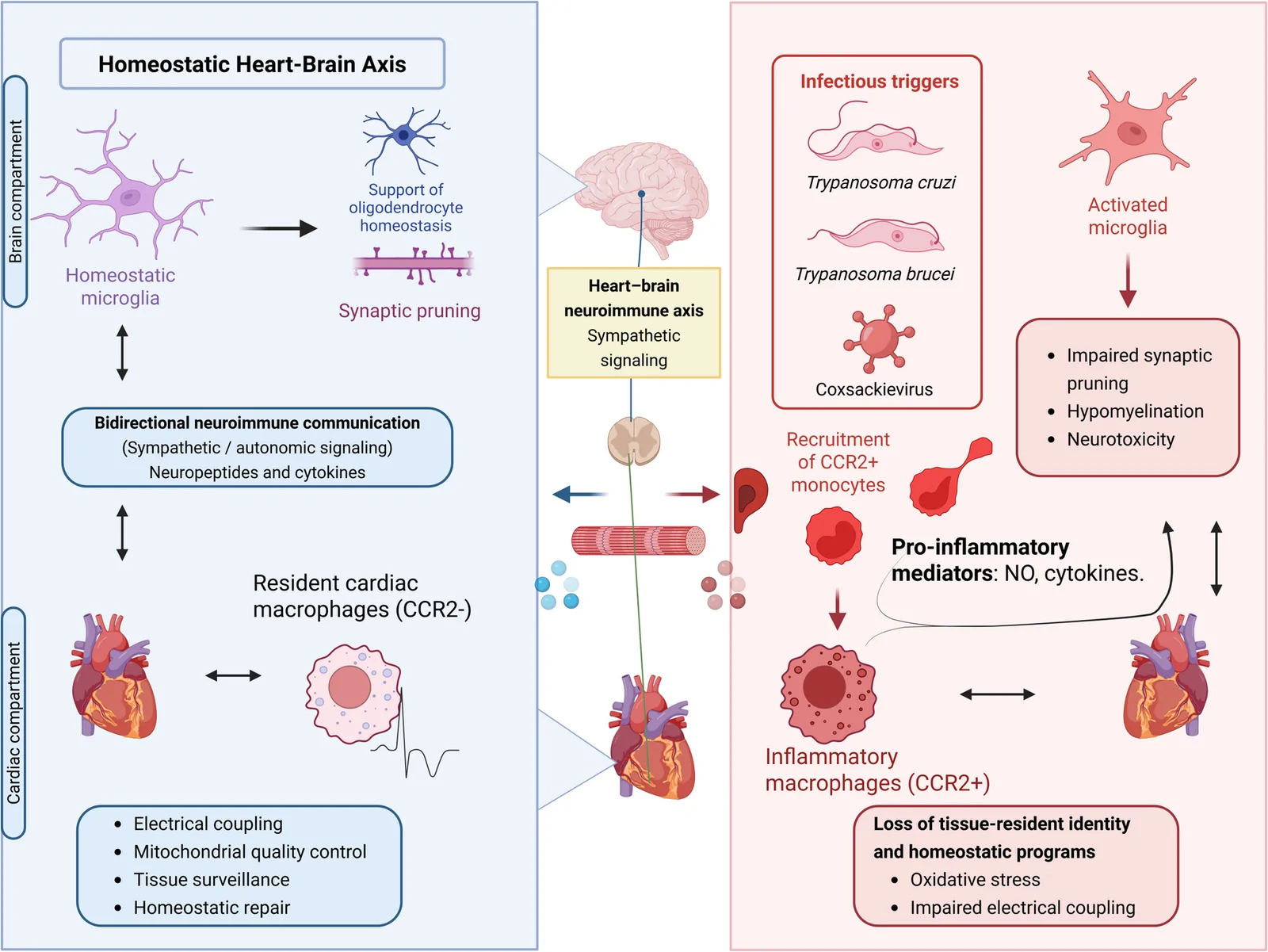
The heart–brain neuroimmune axis in homeostasis and infection. Under homeostatic conditions, resident cardiac macrophages and microglia contribute to organ function through electrical coupling, tissue surveillance, repair programs, synaptic pruning, and support of oligodendrocyte homeostasis. Bidirectional neuroimmune communication involving autonomic signaling, neuropeptides, cytokines, and sympathetic pathways links both compartments. During infection, pathogens such as *Trypanosoma cruzi*, *Trypanosoma brucei*, and Coxsackievirus promote recruitment of CCR2 + monocytes and accumulation of inflammatory macrophages, resulting in increased production of nitric oxide and pro-inflammatory cytokines. These responses are associated with loss of tissue-resident programs, impaired electrical coupling, microglial activation, hypomyelination, defective synaptic remodeling, and neurotoxicity

The discovery that activation of the sympathetic nervous system during cardiac stress triggers the expansion of cardiac macrophages revealed an unexpected principle: the brain orchestrates tissue macrophage responses across distant organs [42]. This heart-brain axis demonstrates how neural circuits influence immunity, and how cardiac macrophages should not be viewed in isolation, but as nodes within a neuroimmune network (Fig. 2).

### Heart Macrophages: Rhythm and Repair

Both healthy mouse and human myocardium harbour distinct populations of macrophages that can be categorised broadly according to their CCR2 expression [43, 44]. Embryonically derived CCR2- macrophages dominate the steady-state myocardium and persist through local self-renewal [44]. Resident cardiac macrophages populate the cardiac interstitium, where they continuously monitor local tissue integrity and promote adaptive remodeling [45]. In contrast, during inflammation, CCR2 + macrophages arise from recruited monocytes, exhibiting enhanced antigen presentation and driving cardiac hypertrophy [46, 47] (Table 1). These populations occupy distinct niches and exert nonredundant effects on cardiac physiology.

**Table 1:** Tissue-resident macrophages: physiological integration and infection

Tissue	Macrophage Population	Homeostatic & Physiological Integration	Infection contexts	References	
Heart	Cardiac macrophages (CCR2- resident)	Electrical coupling via Cx43 gap junctions; cardiomyocyte metabolic support; exopher-mediated mitochondrial clearance.	**Rheumatic valvulitis**: Group A Streptococcus (*S. pyogenes*); **CLP induced sepsis**: Polymicrobial gut derived bacteria; **Viral myocarditis**: Coxsackievirus B3; **Chagas cardiomyopathy**: *Trypanosoma cruzi;* **Myocarditis and cardiac dysfunction**: *Trypanosoma brucei*	[48–52]	
	Cardiac macrophages (CCR2 + recruited)	Antigen presentation; pro-inflammatory cytokine production; initial pathogen containment.	Same as above; dominant during the acute phase.		
Brain	Microglia	Synaptic pruning (C1q/C3-mediated); BBB integrity maintenance; myelin homeostasis; neuronal trophic support.	**Bacterial meningitis**: *S. pneumoniae*,* B.burgdorferi*, *N. meningitidis*; **Viral encephalitis**: HSV-1, ZIKV, HIV; **Protozoans**: *Toxoplasma*, *Plasmodium spp.*, *Leishmania* spp., *Trypanosoma cruzi* and *Trypanosoma brucei;* **Helminths**: *Toxocara* spp, *Angiostrongylus *cantonensis, *Cysticercus cellulosae;* **Fungal**: *Cryptococcus* spp., *Candida albicans*	[53–65]	
	Border-associated macrophages	Gating and restriction of molecular transport across blood-brain and blood-CSF barriers; clearance of cellular debris and metabolic waste from the perivascular spaces; regulation of cerebrospinal fluid production and composition in the choroid plexus	**Bacterial meningitis**: Streptococcus pneumoniae, Group B Streptococcus (GBS); **Protozoans**: Trypanosoma brucei; **Fungal**: *Cryptococcus neoformans*.		
Lung	Alveolar Macrophage (AM)	Surfactant catabolism and recycling; immune tolerance to aeroallergens; GM-CSF-dependent survival.	**Bacterial pneumonia**: *S. pneumoniae*,* K. pneumoniae*,* M. tuberculosis*; **Viral**: *Influenza*, SARS-CoV-2, RSV; **Protozoans**: *Plasmodium* spp., *Toxoplasma*. **Helminths**: *Ascaris spp*,* Toxocara* spp; **Fungal**: *Aspergillus fumigatus*, *Pneumocystis* spp., *Cryptococcus* spp.	[66–73]	
	Interstitial macrophages (IMs)	Parenchymal remodeling; fibroblast crosstalk; perivascular antigen presentation to T cells.	Same as AMs; more prominent in interstitial infections and fibrosis		
Liver	Kupffer Cell (KC)	Systemic blood filtration; clearance of gut-derived LPS; heme-iron recycling; tolerogenic IL-10 production.	**Bacterial sepsis/abscesses**: *E. coli*; *Klebsiella spp*,* Listeria monocytogenes*; **Viral hepatitis**: HBV, HCV; **Protozoans**: *Plasmodium* spp; *Leishmania spp.* **Helminths**: *Schistosoma* spp, *Ascaris* spp; **Fungal**: *Cryptococcus* spp, *Candida albicans*, *Histoplasma capsulatum.*	[74–80]	
Spleen	Red Pulp Macrophages (RPMs)	Erythrocyte senescence sensing; heme-derived iron recycling via HO-1; systemic metabolic filtering.	**Bacteria**: *Salmonella enterica serovar Typhimurium*,* Listeria monocytogenes*; **Viral**: Dengue virus, Adenovirus; **Protozoans**: *Plasmodium* spp, *Leishmania* spp; **Fungal**: *Cryptococcus neoformans*.	[81–86]	
	Marginal & White Pulp (MZM, MMM, TBM)	MZM/MMM: Rapid capture of blood-borne pathogens; antigen delivery to B cells. TBM: Clearance of apoptotic B cells in germinal centers (efferocytosis).	**Encapsulated Bacteria**: *S. pneumoniae*, *E. coli*, *S. aureus*, *Brucella melitensis*; **Viral**: Vesicular stomatitis virus (VSV), Dengue virus; **Fungal**: *Candida albicans*.		
Intestine	Mucosal (MM)	Maintenance of mucosal tolerance; sampling of commensal antigens; TGF-β-mediated inflammatory suppression.	**Bacterial enterocolitis** *Salmonella*,* Helicobacter*,* E. coli*; **Viral gastroenteritis**: Enterovirus; **Protozoans**: *Giardia*, *Entamoeba*,* Cryptosporidium*; **Helminths**: *Trichuris*, *Schistosoma*.	[87–92]	
	Muscularis	Neuro-macrophage crosstalk; BMP2-mediated peristalsis regulation; enteric neuron survival support.	Same as above.		
Skin	Langerhans	Epidermal immunosurveillance; Langerin-mediated antigen uptake; tolerance to skin microbiome.	**Bacterial**: *S. aureus*,* Streptococcus*,* M. leprae*; **Viral**: HSV, VZV, HPV; **Protozoans**: *Leishmania* spp; **Fungal**: *Candida albicans*, *Paracoccidioides brasiliensis.*		[93–98]
	Dermal	Orchestration of wound healing (inflammation/repair transition); VEGF-mediated angiogenesis.	Same as LCs; central in wound infections and diabetic ulcers		

In murine models, resident cardiac macrophages couple electrically to cardiomyocytes through connexin-43-containing gap junctions, thereby influencing atrioventricular conduction [25]. They also clear dysfunctional mitochondria extruded by stressed cardiomyocytes, limiting oxidative injury and directly affecting cardiac performance [99]. Additionally, experimental models show these macrophages serve as specific sentinels for IL-1β, initiating cardioprotective signaling cascades that cardiomyocytes cannot autonomously trigger [100].

The kinetoplastid parasites *Trypanosoma cruzi* and *Trypanosoma brucei* are major causes of infectious heart disease. *T. cruzi* is responsible for the cases of Chagas disease in Latin America, while *T. brucei* causes African trypanosomiasis in rural sub-Saharan Africa [101, 102]. In both settings, cardiac pathology reflects a massive influx of monocyte-derived macrophages. Although controlling the parasite early on depends on this response, sustained activation leads to fibrosis and cardiomyopathy [48, 49]. Indeed, in murine models of *T. cruzi* infection, cardiac macrophages are an important contributor to TGF-β-dependent fibrosis, mediated by metalloproteinases MMP2/MMP9 and PARP1, an intracellular DNA damage sensor which can also modulate gene expression associated with inflammation [103].

Interestingly, infections associated with both cardiac and neurological involvement, such as African trypanosomiasis, Chagas disease, and coxsackievirus infection, frequently present with neurocognitive manifestations [104–106]. In these diseases, neurological dysfunction may be primarily attributed to direct CNS infection and local neuroinflammatory responses [107–109]. However, emerging evidence from non-infectious models suggests that cardiac injury can influence microglial function through autonomic and neuroimmune pathways [110, 111]. Whether similar heart-brain communication mechanisms contribute to neurological manifestations during infectious diseases remains to be established. Given the prominent participation of inflammatory CCR2 + monocyte/macrophage populations in both cardiac and cerebral pathology [112, 113], investigating the heart-brain axis in these infections represents an important avenue for future research.

Taken together, cardiac macrophages exemplify how tissue-resident macrophages are integrated into organ physiology. Their functions extend beyond antimicrobial defense to the regulation of electrical conduction, mitochondrial quality control, and tissue remodeling. During infection, disruption of these homeostatic programs contributes not only to pathogen control but also to long-term alterations in cardiac performance, illustrating how resident macrophages translate inflammatory signals into physiological outcomes.

### Central Nervous System Resident Macrophages: Microglia and Border-Associated Macrophages

The central nervous system (CNS) harbors two major populations of tissue-resident macrophages that occupy distinct anatomical niches and perform complementary functions in immune surveillance. Parenchymal microglia are distributed throughout the brain and spinal cord parenchyma, whereas CNS-associated macrophages (CAMs), also referred to as border-associated macrophages (BAMs), reside at CNS interfaces, including the leptomeninges, perivascular spaces, and choroid plexus [53, 114, 115]. Microglia remain largely maintained through local self-renewal with minimal contribution from circulating monocytes under homeostatic conditions. Microglia express markers such as P2Y12 [116], TMEM119 [117], and SALL1 [118], while BAMs are characterized by the expression of CD163 [119], LYVE1 [120], and MRC1 [121]. Together, these resident macrophage populations form an integrated surveillance network that maintains CNS homeostasis and coordinates responses to infection, injury, and neurodegeneration.

Lineage-tracing studies in mice have demonstrated that microglia originate from early yolk-sac macrophage progenitors generated during embryonic hematopoiesis and subsequently maintain themselves largely through local self-renewal throughout adult life [2, 7, 8]. In contrast to microglia, BAMs are strategically positioned at the CNS–blood and CNS–cerebrospinal fluid interfaces, where they act as sentinels of incoming environmental and circulating signals [54]. Studies have demonstrated that these macrophages actively participate in antigen presentation, leukocyte recruitment, regulation of vascular inflammation, and host defense against neuroinvasive pathogens [53, 121]. Owing to their localization, BAMs are among the first immune cells to encounter blood-borne microorganisms attempting to access the CNS.

Under homeostasis, microglia are hypermobile surveyors that continuously extend and retract their processes [122]. Through complement-mediated mechanisms, they prune weak synapses, refining neural circuits [123]. Neurons tag redundant synapses with C1q and C3, and the microglia’s CR3 (CD11b/CD18) recognizes these tags, triggering the phagocytosis of tagged synapses [123, 124]. This pruning is essential for learning, memory, and circuit refinement during development.

Recent studies have identified disease-associated microglia (DAM), which were initially described in Alzheimer’s disease but are now recognized across diverse infectious and inflammatory conditions [125, 126] (Fig. 2). DAM profiles are characterized by the downregulation of homeostatic genes and the upregulation of pathways associated with lipid metabolism, phagocytosis, and inflammatory signaling [125]. In experimental models of SARS-CoV-2 infection, microglia acquire a neuroinflammatory phenotype associated with myelin loss and impaired hippocampal neurogenesis, changes that correlate with cognitive deficits [127]. Complementing these mechanistic observations, neuroimaging studies in humans have identified structural brain alterations following COVID-19 [128]. It should be noted that recent efforts to harmonize microglia state nomenclature across disease conditions have highlighted both shared and disease-specific features of microglial activation in both human and rodent datasets [129, 130].

During CNS infection, pathogens may exploit these cells through distinct mechanisms depending on their route of entry and tissue tropism. Monocyte depletion with clodronate liposomes, which under conditions of blood-brain barrier (BBB) disruption may also affect resident CNS macrophage populations, reduced fungal burden and meningitis severity [131], suggesting a pathogenic role for myeloid cells in facilitating CNS invasion, though the specific contribution of peripheral monocytes versus resident CNS macrophages cannot be fully resolved with this approach. More broadly, CNS infiltrating myeloid cells are phenotypically and functionally distinct from parenchymal microglia. They lack the homeostatic microglial signature, including P2RY12 expression [55], and are typically cleared following resolution of the acute phase [132].

On the other hand, chronic neuroinfection by the protozoans *Toxoplasma gondii* and *Leishmania* spp triggers localized production of anti-inflammatory loops (IL-10 and TGF-β) by parenchymal microglia to restrain secondary neurodegeneration [55, 56]. Following successful pharmacological clearance of *T. brucei*, recruited inflammatory monocytes are rapidly cleared via apoptosis, whereas resident BAMs exhibit a lingering inflammatory signature that permanently reshapes their proteogenomic landscape [57].

Dysfunctional microglia are also heavily involved in the pathophysiology of heart damage. During heart failure, there is a significant increase in vessel-associated microglia and a decrease in parenchymal microglia [133]. Once these microglia are activated and recruited to the blood vessels, they upregulate the production of pro-inflammatory cytokines, most notably TNFα [110, 133]. This binding initiates a neuroinflammatory cascade that contributes to cardiac remodeling, autonomic imbalance, and cognitive deficits in patients with heart disease.

Collectively, CNS resident macrophages occupy strategically distinct niches that allow them to coordinate immune surveillance while preserving neural function. During infection, alterations in these highly specialized programs can persist beyond pathogen clearance, linking neuroinflammation to long-term cognitive and neurological sequelae.

### Connecting Brain and Heart: A Bidirectional Neuroimmune Circuit

The heart-brain axis operates bidirectionally. Cardiac stress activates brain-coordinated immune responses via sympathetic outputs, and CNS infection can trigger systemic inflammation that affects cardiac function [134, 135] (Fig. 2). Severe encephalitis often presents with cardiac arrhythmias and dysfunction, which are potentially mediated by altered autonomic tone and systemic cytokine release [136, 137]. Understanding this bidirectional communication may inform therapies that modulate neuroimmune circuits to limit cardiac and neurological damage during severe infections. Although heart-brain communication has been investigated in cardiovascular and neurodegenerative disorders, its contribution to infectious diseases that simultaneously affect cardiac and neural tissues remains poorly understood. Therefore, extrapolations to infections such as trypanosomiasis and enteroviral diseases should currently be regarded as hypothesis-generating mechanisms.

This bidirectional communication presents therapeutic opportunities. Vagal nerve stimulation has been shown to limit the generation of monocyte-derived inflammatory CCRL2 + macrophages during heart failure [112], thereby reducing inflammatory amplification within the myocardium. Separately, vagal stimulation has been shown to alter the function of cardiac-resident macrophage populations in a manner that supports tissue homeostasis [138]. Beta-blockers, which are traditionally prescribed for rate control, also modulate macrophage activation by blocking adrenergic receptors on immune cells [139]. Emerging evidence suggests that neuromodulation strategies could serve as macrophage-targeted therapies, dampening harmful inflammation while preserving homeostatic functions across the heart-brain circuit (Fig. 2).

Several neuropeptides that were originally discovered in the brain have profound cardiovascular effects. Cortistatin, substance P, and neuropeptide Y are now known to be widely expressed in the cardiovascular system, where they protect the heart against ischemia, vascular calcification, and inflammation [140, 141]. Though they have been studied less in the context of infection, modulating the heart-brain axis using endogenous neuropeptides like cortistatin is a highly promising therapeutic approach for protecting the heart during and after severe infections.

The heart-brain axis illustrates how tissue-resident macrophages participate in physiological circuits that extend beyond individual organs. By integrating neural, immune, and tissue-derived signals, macrophages can propagate the consequences of infection across organ systems, linking local inflammation to systemic dysfunction.

## Splenic and Hepatic Macrophages: Iron Metabolism and Systemic Pathogen Control

**Fig. 3:**
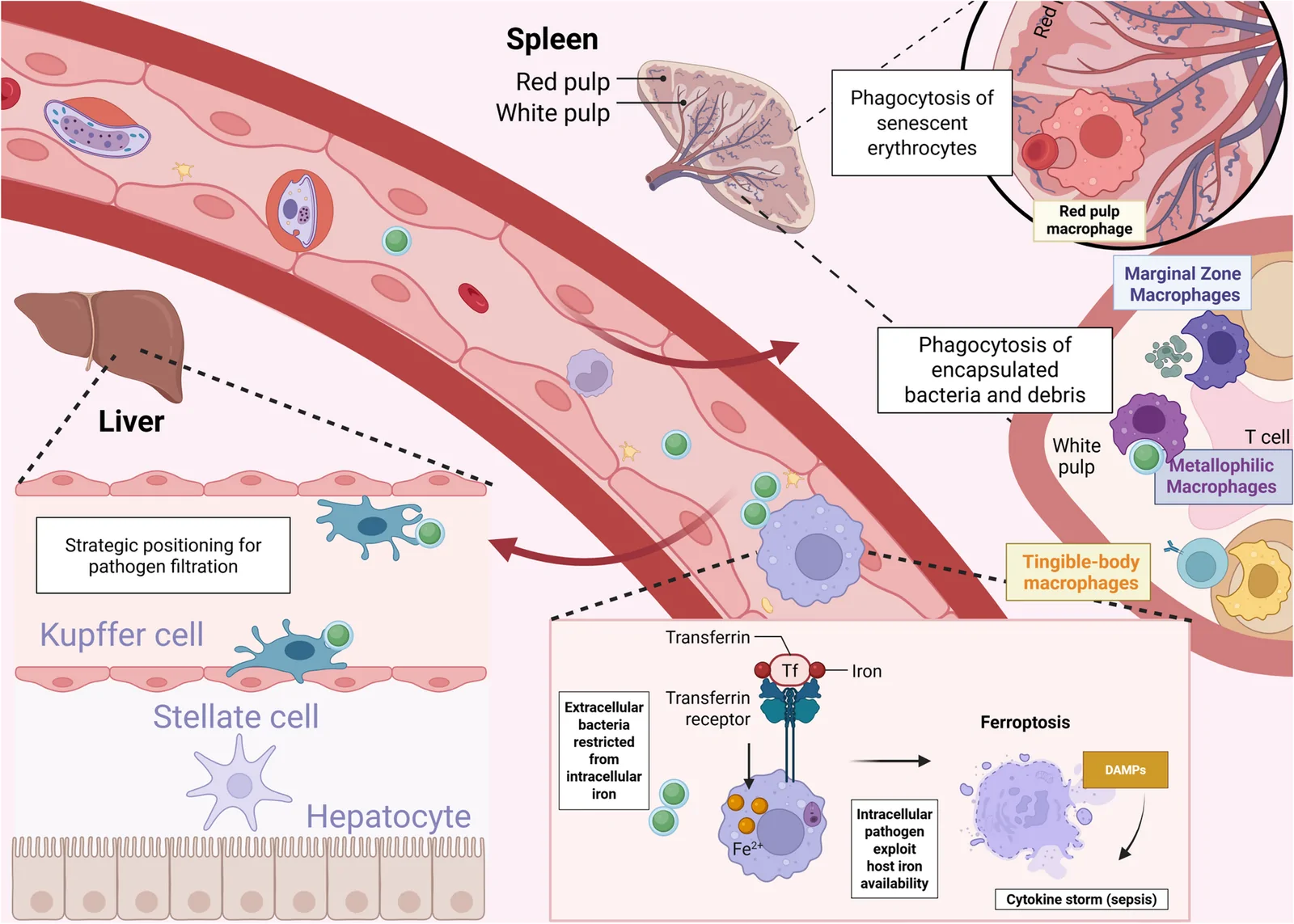
The liver–spleen axis: systemic filtration, iron recycling, and immunity. The spleen and liver function as interconnected hubs for blood filtration and iron homeostasis. Red pulp macrophages recycle iron derived from senescent erythrocytes, whereas marginal zone and metallophilic macrophages capture blood-borne pathogens and debris. Within the liver, Kupffer cells filter portal blood and contribute to iron handling and immune surveillance. Iron sequestration within macrophages restricts extracellular pathogen access to this essential nutrient, whereas intracellular pathogens may exploit host iron availability. Excessive iron accumulation can promote ferroptosis, leading to macrophage loss, release of damage-associated molecular patterns (DAMPs), and amplification of systemic inflammation

The heart-brain axis coordinates localized tissue responses through neural circuits, while the liver-spleen axis governs systemic immunity through iron. Both organs filter blood, harbor self-renewing embryonic macrophages, and manipulate iron availability for metabolic necessity and as an antimicrobial strategy [142–144] (Fig. 3). Their coordinated function is especially critical during blood-borne infections, such as malaria, where the lifecycle of *Plasmodium*, the destruction of erythrocytes, and the immune response converge on iron homeostasis (Table 1).

### Splenic Macrophages: Filtration and Iron Recycling

The spleen is a complex mosaic of immune niches. Preclinical evidence outlines how distinct populations divide responsibilities for systemic filtration, iron recycling, and rapid pathogen capture [145, 146]. The human spleen clears an estimated two million senescent erythrocytes per second [147], generating a continuous flux of heme-derived iron that must be efficiently processed and returned to the circulation to sustain erythropoiesis, a task that falls almost entirely to red pulp macrophages (RPMs) [148].

RPMs are established predominantly from embryonic precursors and maintained largely through local self-renewal under homeostatic conditions [149]. Their identity in mice is governed by the transcription factor SPI-C, induced by the very heme they ingest [150]. Other markers include F4/80^high^ [151], CD163 [150] and VCAM-1 [150].

RPMs discriminate between viable and senescent erythrocytes through a finely tuned balance of opposing surface signals. Under homeostatic conditions, CD47 expressed on healthy erythrocytes binds to the inhibitory receptor SIRPα on RPMs, initiating an intracellular signaling cascade that recruits SHP-1 and SHP-2 phosphatases to actively suppress actomyosin-dependent engulfment [152, 153]. As erythrocytes age or undergo oxidative damage, CD47 becomes progressively depleted or structurally altered on the erythrocyte surface, removing the requisite threshold for SIRPα ligation [154, 155]. Concurrently, the exposure of phosphatidylserine on the outer leaflet of the senescent erythrocyte membrane presents an inductive signal that directly engages activating receptors on RPMs, including TIM4 and MerTK [156, 157]. The net result of this diminished inhibitory signaling on the erythrocyte and elevated receptor engagement on the macrophage is the targeted clearance of damaged cells.

Upon phagocytosis, they degrade hemoglobin and recycle heme-derived iron via heme oxygenase-1 (HO-1), returning iron to circulation to support erythropoiesis [158]. In murine models of systemic infection or malaria, the capacity of RPMs to sequester iron increases responsiveness to hepcidin [159, 160]. Hepcidin is a liver-derived hormone that promotes the internalisation and degradation of ferroportin, the principal cellular iron exporter. This restricts iron release and limits pathogens’ access to this essential nutrient [161]. Also, in murine infection with *Plasmodium chabaudi*, a rodent malaria parasite commonly used as an experimental model of blood-stage malaria, inflammatory monocytes replenish depleted RPM populations, aimed to control parasite recrudescence and prevent persistent infection [81, 162].

In mice, marginal zone macrophages (MZMs) and metallophilic macrophages (MMMs) function as primary sentinels for blood-borne pathogens [163]. Positioned at the interface between the white and red pulp, murine MZMs are identified by expression of MARCO [164] and SIGN-R1/CD209 [165]. These macrophages occupy a specialized anatomical niche that differs substantially from splenic macrophage organization in humans [166]. In mice, MZMs are uniquely equipped to capture blood-borne pathogens, particularly encapsulated bacteria [167, 168]. Their high expression of pattern recognition receptors, including scavenger receptors (MARCO) [164], C-type lectins (SIGN-R1) [165], and Toll-like receptors (TLR1/2) [169], allows them to trap pathogens from the high-velocity blood flow and initiate a rapid immune response.

In mice, MMMs are distinguished by CD163 and CD169+ (Siglec-1) expression, form a physical barrier at the marginal sinus and excel at trapping lymph-borne bacteria and apoptotic debris [170, 171]. While human splenic architectures lack a distinct marginal sinus counterpart, equivalent macrophage subsets line the open circulation of the human red pulp to execute overlapping filtration tasks [166].

During infection, preclinical models and human observational data converge to show that the splenic macrophage network faces extreme functional demands. In malaria, the RPM population must expand massively to handle the burden of parasite-infected red cells [172, 173]. Paradoxically, this expansion depends on monocyte recruitment, suggesting that the resident embryonically derived RPM compartment alone is unable to expand sufficiently to accommodate the extreme erythrophagocytic demands imposed by malaria, thereby necessitating recruitment of monocyte-derived macrophages [81].

In chronic infections, including visceral leishmaniasis and HIV, parasites and viruses exploit splenic macrophages as replicative niches, subverting antimicrobial programs through phagolysosomal fusion inhibition and STAT1 signaling blockade [174, 175]. Resolution requires restoration of SPI-C+ RPMs, whether through monocyte differentiation or local proliferation, as prerequisites for systemic metabolic health, including anemia of inflammation resolution and iron trafficking restoration [176].

Furthermore, less characterized, splenic tingible-body macrophages (TBMs), located within germinal centers (CD68+, high phagocytic capacity), continuously clear apoptotic B cells generated during germinal center reactions, where extensive proliferation and affinity maturation produce large numbers of non-selected or autoreactive clones [177, 178].

The splenic macrophage network illustrates how tissue specialization enables distinct resident populations to coordinate blood filtration, iron recycling, antigen capture, and adaptive immune regulation. Infection places extraordinary demands on these functions, forcing macrophages to balance pathogen control with preservation of systemic metabolic homeostasis.

### Kupffer Cells: The Liver’s Filtration System

The liver is continuously exposed to dietary antigens, microbial products, and metabolites arriving from the gut via the portal vein [179]. Kupffer cells (KCs) are ideally positioned within hepatic sinusoids to filter blood, catching gut-derived pathogens and toxins from the portal circulation before they can enter the systemic blood [180] (Table 1). KCs disturbance during inflammation triggers structural vascular alterations and systemic pathogen susceptibility [181].

Fate-mapping studies in mice indicate that KCs derive predominantly from yolk-sac erythromyeloid progenitors and are maintained largely independently of circulating monocytes under homeostatic conditions [182, 183]. Niche signaling drives expression of master transcription factors: ID3 [184, 185], LXR-α [184, 185] and SPI-C [185], which collectively govern definitive KC identity. It is worth noting that while ID3 acts as a core regulator of the hepatic myeloid lineage, its functional requirement was characterized by Bonnardel and colleagues [184] specifically within the context of monocyte-derived macrophage repopulation and niche engraftment following resident cell depletion, whereas its steady-state maintenance operates in concert with embryo-derived ancestral programs [185]. Defining murine surface markers CLEC4F and CRIg (Complement Receptor of the Immunoglobulin superfamily) enable bacterial capture from fast-flowing blood under high shear stress [184, 186]. However, they preferentially induce tolerogenic programs, producing IL-10 and prostaglandins that dampen excessive immune activation [187]. This tolerogenic bias proves essential for preventing chronic liver inflammation in response to gut-derived signals.

During infection, KCs encounter distinct challenges depending on pathogen tropism. During the hepatic stage of malaria, *Plasmodium* sporozoites directly interact with KCs, making these cells one of the first immune barriers encountered before hepatocyte invasion [188, 189]. In schistosomiasis, eggs trapped within portal venules induce the recruitment of monocyte-derived macrophages and the formation of granulomas that sequester egg antigens and limit tissue injury [190]. Although initially protective, persistent granulomatous inflammation promotes periportal fibrosis and progressive remodeling. After the resolution of infection, monocyte-derived macrophages engraft in the liver, assume a KC-like profile and co-exist with embryonic KCs in the long-term [191]. The co-existence of embryonic KCs and monocyte-derived KC-like cells following infection resolution [191] provides a naturally occurring example of monocyte-to-resident convergence in which monocyte-derived cells progressively acquire the KC transcriptional and epigenetic signature under sustained hepatic niche signals, though whether they fully recapitulate embryonic KC functions remains an open question.

Embryonic KCs’ primary non-immune function is iron recycling. They phagocytose senescent erythrocytes, degrade hemoglobin, and recycle iron to bone marrow for erythropoiesis. Ferroportin (iron exporter) expression is regulated by systemic hepcidin hormone [192]. A major finding in this field is the role of ferroptosis, an iron-dependent form of cell death driven by lipid peroxidation [193]. Experimental models of malaria indicate that the uptake of hemozoin by KCs triggers lipid peroxidation and ferroptosis, leading to acute depletion of these resident cells [74]. This loss of KCs impairs the host’s ability to clear secondary infections, explaining why malaria patients exhibit heightened vulnerability to fatal bacteremia [74]. In experimental visceral leishmaniasis, *Leishmania donovani* infects KCs and exploits this machinery, activating the Nrf2 antioxidant pathway to prevent its own death by ferroptosis, maintaining a permissive niche for replication [194, 195]. The parasite’s ability to subvert the KC population reflects the resilience of niche-educated macrophage identity, while also illustrating how that same identity can be turned against the host [75].

The spleen and liver are functionally linked through a continuous cycle of erythrocyte turnover and iron trafficking [142, 196]. Iron recovered by splenic and hepatic macrophages is exported through ferroportin and enters systemic circulation, while hepatic hepcidin production regulates iron release from both compartments. During malaria and other hemolytic infections, increased erythrocyte destruction alters this balance, triggering coordinated adaptations in both organs [197]. Thus, the liver-spleen axis represents an integrated physiological circuit in which macrophages coordinate systemic iron availability, blood filtration, and antimicrobial defense. Together, splenic and hepatic macrophages demonstrate how tissue-resident macrophages coordinate organism-wide metabolic adaptations during infection. Their ability to regulate iron availability, erythrocyte turnover, and pathogen clearance positions them as central mediators linking immunity to systemic physiology.

However, these systemic immune challenges differ fundamentally from those faced at barrier tissues, where the constant presence of microbial communities and environmental antigens requires exquisite discrimination between harmless and harmful.

## Barrier Macrophages: Constant Exposure, Selective Response

**Fig. 4:**
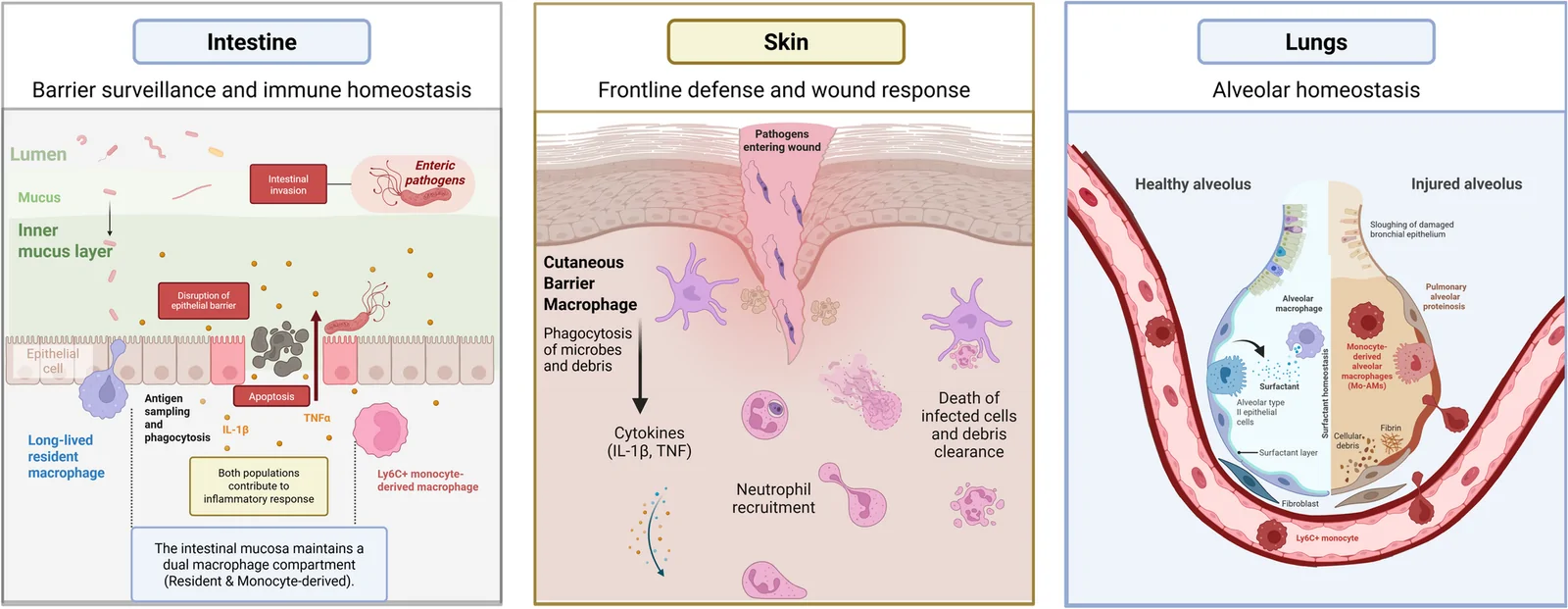
Barrier tissue macrophages coordinate homeostasis and host defense in the intestine, skin, and lungs. Barrier-associated macrophages integrate tissue maintenance with antimicrobial defense. In the intestine, long-lived resident macrophages continuously sample luminal antigens, remove apoptotic cells, and preserve epithelial integrity, whereas recruited Ly6C+ monocyte-derived macrophages contribute to inflammatory responses following epithelial disruption and microbial invasion. In the skin, cutaneous macrophages participate in wound surveillance, phagocytosis of pathogens and cellular debris, cytokine production, and recruitment of additional immune cells during tissue injury. In the lungs, alveolar macrophages maintain surfactant homeostasis and alveolar function under steady-state conditions, whereas severe injury promotes recruitment of monocyte-derived alveolar macrophages and accumulation of cellular debris, processes associated with loss of alveolar homeostasis and pulmonary alveolar proteinosis. Together, these populations illustrate how barrier macrophages balance tissue tolerance with rapid antimicrobial defense

While heart-brain circuits coordinate distant organs and liver-spleen networks govern systemic immunity, a third challenge confronts barrier tissues: maintaining tolerance to commensal communities and environmental antigens, responding decisively to invading pathogens, and limiting collateral tissue damage that would compromise barrier integrity (Fig. 4). The lung, intestine, and skin each harbor specialized macrophage populations that have evolved exquisite mechanisms to distinguish harmless from harmful, yet infection relentlessly tests these discriminatory systems [198, 199] (Table 1). We examine how barrier macrophages integrate metabolic, microbial, and damage signals to adjudicate this fundamental immunological decision.

### Alveolar Macrophages: Sentinels of the Airspace

The lung presents unique immunological challenges: constant exposure to airborne particles, commensals, and pathogens necessitates high activation thresholds balanced with rapid response capacity. Two principal populations dominate: alveolar macrophages (AMs) residing freely in the alveolar airspace, and interstitial macrophages (IMs) embedded within lung parenchyma [200, 201] (Table 1; Fig. 1).

Murine fate-mapping studies have shown that the alveolar macrophage pool is established through successive developmental waves [202, 203]. EMP-derived precursors initially seed the fetal lung and establish an early resident population. During the perinatal period, this pool is progressively supplemented by cells arising from fetal hematopoiesis, though the precise sequence and relative contribution of embryonic intermediates, including pMacs and fetal monocytes, linking EMPs to mature alveolar macrophages remains an area of active investigation and likely differs among tissues and developmental stages [9, 10, 204]. Full acquisition of the AM transcriptional identity, governed by PPAR-γ and dependent on GM-CSF secreted by alveolar type II epithelial cells, occurs postnatally as the alveolar niche matures [37, 205]. GM-CSF is also necessary to sustain AM survival and self-maintenance throughout adult life [205]. The resulting AM population is identified in mice by co-expression of Siglec-F [206], CD11c, CD64, and MerTK [66], and in humans by CD206 [207] and CD169 [208] among other markers [208].

AMs constitute the primary catabolic machinery for surfactant recycling, ingesting and degrading surfactant lipids via lysosomal pathways regulated by PPAR-γ and Bach2 [209, 210]. Since the alveolar space contains abundant lipids (pulmonary surfactant preventing alveolar collapse), this adipogenic programming proves functional. Absence or dysfunction of this pathway precipitates pulmonary alveolar proteinosis, characterized by lungs drowning in their own secretions [209]. AMs maintain exceptionally high activation thresholds under steady-state conditions, actively suppressing immune responses through IL-10, TGF-β, and inhibitory receptor signaling [211]. This restraint maintains tolerance despite constant particulate and microbial challenge.

Severe influenza infection in mice resets the alveolar compartment. Resident embryonic AMs prove highly susceptible to influenza-induced necroptosis and apoptosis. As they die, Ly6C+ inflammatory monocytes are recruited, differentiating into monocyte-derived AMs (Mo-AMs) [67, 212]. While Mo-AMs eventually upregulate resident markers (Siglec-F, CD11c), they are not functionally identical to original residents. Transcriptomic analyses of these murine populations reveal Mo-AMs’ heightened propensity for pro-inflammatory cytokine production (IL-6, TNF) and reduced tissue repair efficiency compared to resident AMs [213]. Whether this monocyte dominance permanently predisposes the host to secondary bacterial pneumonia or instead confers heterologous protection via trained immunity remains highly context-dependent and incompletely resolved [66, 68, 214]. This transcriptional convergence illustrates the capacity of monocyte-derived cells to progressively acquire niche-driven resident-like programs under sustained environmental signals, while also demonstrating that such convergence is incomplete and does not fully recapitulate the functional identity of embryonically established alveolar macrophages.

During pulmonary *Mycobacterium tuberculosis* infection, alveolar macrophages exhibit distinct functional alterations. The bacilli evade microbicidal mechanisms within the phagolysosome and drive the accumulation of lipid bodies within the macrophage cytoplasm, resulting in a foamy macrophage phenotype [69]. Therapeutic targeting of this pathway using metformin to activate AMPK restores mitochondrial fitness and enhances intracellular bacterial clearance, highlighting the therapeutic potential of metabolic host-directed interventions [215, 216].

In an elegant study, Chakarov and colleagues revealed that IMs are organized into segregated sub-populations defined by the expression of LYVE1, CX3CR1, and MHC-II [217]. The LYVE1^high^ MHC-II^low^ IM subset is localized primarily in perivascular and perineural niches; in contrast, the neighboring LYVE1^low^ MHC-II^high^ IM pool is continuous with systemic hematopoiesis. Both populations rely on steady-state influx and terminal imprinting of circulating Ly6C^high^ monocytes [217]. During pulmonary infections, the MHC-II^high^ sub-population expands rapidly to coordinate antigen presentation and T cell priming, but can persistently drive pathological pulmonary fibrosis if chronically dysregulated. Additional studies have also identified CD206 expression as an important marker distinguishing specific interstitial macrophage subsets, particularly those associated with perivascular niches and tissue repair [218].

Alveolar macrophages illustrate the intimate relationship between tissue metabolism and immune function. Their specialized role in surfactant turnover establishes a physiological program that constrains inflammatory responses under homeostatic conditions. Infection can durably alter this program through replacement of embryonic residents by monocyte-derived macrophages, demonstrating how transient inflammatory events may reconfigure pulmonary physiology long after pathogen clearance.

### Intestinal Macrophages: Mucosal Peacekeepers in a Microbial World

The gut mucosa represents the most complex immunological environment in the body, requiring robust tolerance to dietary components and commensals alongside a prompt activation threshold against pathogens. Two anatomically and functionally distinct lineages, mucosal macrophages (lamina propria) and muscularis macrophages, execute these tasks [219].

Recent single cell dataset has fundamentally updated the classical view of a uniform, monocyte-dependent renewal cascade within the mucosa, revealing instead a dual-compartment organization [220]. The mucosal lamina propria harbors a stable, long-lived subset of tissue-resident macrophages characterized by the expression of TIM4, CD163 and CD4 [220] (although typically associated with lymphocytes, CD4 can be used as a maturation marker for gut macrophages). Key functions include clearance of apoptotic cells [221], maintenance of the intestinal crypts homeostasis [222], and supporting neuroimmune interactions [33]. Co-existing with this stable population is a highly dynamic, short-lived macrophage compartment located directly beneath the epithelial basement membrane. This sub-population undergoes continuous, rapid replenishment from circulating Ly6C^high^ monocytes driven by steady-state CCR2–CCL2 signaling trajectories [223].

Regardless of their distinct lifespans and ontogenic trajectories, the local intestinal niche imposes a strict regulatory program on mucosal populations [224]. In both human biopsies and rodent tissues, entering monocytes downregulate inflammatory receptors (CD14) while upregulating tolerogenic markers (CX3CR1^high^, IL-10+) [219, 225]. Among the niche-derived signals that maintain mucosal macrophage tolerance, aryl hydrocarbon receptor (AhR) activation by dietary tryptophan metabolites and microbial indoles has emerged as a key pathway suppressing inflammatory activation in the lamina propria [226, 227]. This specialized imprinting ensures that both long-lived and monocyte-derived mucosal macrophages maintain a high activation threshold, retaining potent phagocytic capacity while remaining largely refractory to pattern recognition receptor-induced cytokine storms during homeostatic surveillance.

During bacterial enterocolitis caused by invasive pathogens such as *Salmonella enterica* or *Campylobacter jejuni*, virulence factors trigger inflammasome activation within mucosal macrophages, leading to the release of active IL-1β [228, 229]. While recruited monocytes differentiate into inflammatory macrophages that express elevated levels of TNF and iNOS, evidence from inflammatory bowel disease and colitis models indicates that both resident and recruited myeloid sub-populations contribute to active pathology and subsequent resolution in a context-dependent manner [230, 231].

Deep within the muscularis externa, self-renewing muscularis macrophages intimately associate with the myenteric plexus [33, 232]. This neuroimmune circuit is governed by reciprocal signaling, where enteric neurons supply essential CSF-1 and macrophages return BMP2 to regulate peristalsis [233, 234]. These resident cells are also phenotypically marked by TIM4 and MHC-II [235–238], playing fundamental roles in the maintenance of intestinal movement, tissue homeostasis, and enteric nervous system integrity. The functional importance of this axis becomes critically evident during pathological challenges. During *Salmonella* infection, for instance, the activation of β2-adrenergic receptors on these muscularis macrophages induces a tissue-protective program that effectively limits neuronal apoptosis and prevents post-infectious dysmotility [236]. In contrast, during enteric helminth models, alternative activation programs are driven primarily by T-cell-derived cytokines; these pathways support localized tissue protection and mucosal remodeling independently of direct muscularis macrophage-mediated changes in peristalsis [237]. Whether this exact characteristic translates completely to human physiology remains to be fully elucidated, but recent single-cell transcriptomic analyses show promising parallels across species [239]. Preclinical models of intestinal infection continue to map these precise pathways [87], shedding light on how intracellular persistence within macrophage niches shapes chronic post-infectious sequelae [88, 240].

Intestinal macrophages operate at the intersection of immunity, microbial ecology, and neurophysiology. By simultaneously regulating tolerance, epithelial integrity, and enteric neuronal function, these cells preserve gastrointestinal homeostasis despite constant microbial exposure. Infection disrupts these interactions, often generating persistent alterations in motility and mucosal function that outlast the acute inflammatory response.

### Skin Macrophages: Dermal Defenders and Wound Healers

The skin is the body’s largest organ and its primary barrier against the external world. Within this layered interface, two distinct macrophage populations reside: Langerhans cells (LCs) in the epidermis and dermal macrophages (DMs) in the underlying dermis [241]. While both contribute to cutaneous immunity, they occupy fundamentally different niches and respond to infection in divergent ways [242].

The formal demonstration by Hoeffel and colleagues [243] that LCs arise from fetal liver precursors and self-maintain throughout adult life independently of bone marrow hematopoiesis provided early and influential evidence for embryonic seeding as a principle of tissue-resident immune cell biology. This work, alongside contemporaneous discoveries establishing the embryonic origin of microglia [2], contributed to a conceptual shift away from the view that all tissue macrophages are continuously replenished by circulating monocytes.

Residing in epidermis, they extend dendrites through tight junctions sampling the surface. Upon activation, they can migrate to draining lymph nodes priming T cells, a capability most TRMs lack. LC development depends on TGF-β and IL-34 (keratinocyte-secreted CSF1R ligand), governed by transcription factors Runx3 and ID2 [241, 244].

Under homeostasis, LCs maintain tolerance, continuously sampling keratinocyte-derived self-antigens and commensal bacteria, presenting them tolerogenically to prevent autoimmunity and maintain peaceful skin microbiome coexistence [245]. During *Candida* and *Malassezia* infection, however, LCs shift to immunogenic antigen presentation: Langerin binds fungal β-glucan, triggering activation and lymph node migration where they prime Th17 responses essential for fungal clearance [93, 246, 247].

DMs represent a mixture of yolk-sac residents and monocyte-derived cells, residing throughout dermis (particularly around blood vessels and hair follicles). Dermal macrophages are established through successive developmental contributions: fetal-derived populations seed the dermis early and persist with relatively slow turnover, while monocyte-derived cells are continuously recruited to supplement or partially replace them, particularly in perivascular niches. Importantly, fetal-derived and monocyte-derived dermal subsets differ in their longevity, inflammatory responsiveness, and regenerative behavior following tissue injury [248, 249]. Heterogeneously marked by CX3CR1, F4/80, Lyve1, and MHC-II [250, 251], they are governed by transcription factors MAFB and c-MAF [252]. Fetal-derived dermal macrophages exhibit greater longevity and stronger integration into tissue repair and neuroimmune circuits, whereas monocyte-derived populations display higher turnover and inflammatory responsiveness. Thus, developmental origin contributes directly to the functional specialization of dermal macrophage subsets within the skin [253, 254].

DMs excel at wound healing, unfolding through overlapping inflammation, proliferation, and remodeling phases. Immediately post-injury, DAMP-activated resident DMs produce inflammatory cytokines (TNF, IL-1β) and chemokines (CCL2, CXCL8) recruiting neutrophils and additional monocytes [255]. As inflammation resolves, macrophages transition to reparative phenotypes producing growth factors (VEGF, PDGF, TGF-β), matrix metalloproteinases (tissue remodeling), and arginase-1 (collagen synthesis). IL-4 and IL-13 from T cells, mast cells, and eosinophils drive this transition [256].

However, infection disrupts this orchestrated repair. *Staphylococcus aureus*, the most common agent of wound infection, triggers robust macrophage activation via TLR2 and inflammasome pathways. Infected macrophages produce IL-1β recruiting neutrophils, initiating abscess formation [257].

In cutaneous leishmaniasis, parasites target DMs. Neutrophils phagocytose parasites but cannot kill them [258]. DMs then engulf dying infected neutrophils via efferocytosis (phagocytosis without inflammation triggering). This silent entry prevents macrophage activation, delivering parasites safely into replicative niches without triggering robust immunity [258].

Post-infectious scarring also reflects macrophage dysfunction. Following severe bacterial or viral skin infections (herpes zoster, *S. aureus* cellulitis), persistent dermal macrophages produce excessive TGF-β1 and PDGF, driving fibroblast proliferation and extracellular matrix deposition [259–261].

Recent studies have revealed that a subset of embryonically derived dermal macrophages is positioned in close association with cutaneous sensory neurons, forming a specialized neuroimmune unit within the skin [254, 262]. Neuron-derived trophic signals contribute to macrophage survival and maintenance of anti-inflammatory transcriptional programs, while dermal macrophages support local tissue repair and vascular homeostasis. Disruption of this bidirectional communication may impair wound healing and regenerative responses [263].

This mechanism may contribute to persistent skin pathology observed in disorders such as leprosy. During *Mycobacterium leprae* infection, the bacillus preferentially infects dermal macrophages and Schwann cells, inducing extensive metabolic reprogramming characterized by lipid accumulation and extensive demyelination [264, 265]. Infected macrophages contribute to bacterial persistence and participate in immune evasion by downregulating CD8 + T cell-mediated cytotoxicity [266]. This neuroimmune decoupling impairs the coordination of localized wound healing and microvascular homeostasis, providing an explanation for why certain chronic infectious and neurodegenerative conditions leave persistent, non-healing cutaneous ulcerations independently of direct macrophage infection.

Skin macrophages demonstrate how tissue-resident immune cells integrate antimicrobial defense with tissue regeneration. Their ability to transition between inflammatory and reparative programs determines not only pathogen clearance but also the quality of tissue restoration. When these regulatory mechanisms fail, infection may leave a lasting physiological imprint in the form of fibrosis, scarring, or chronic inflammatory disease.

Although neuroimmune communication has traditionally been studied within the central nervous system, a growing body of evidence indicates that tissue-resident macrophages across peripheral organs are also embedded within neural circuits that continuously shape their behavior [249, 262]. Macrophages receive neurotransmitter and neuropeptide signals that tune activation thresholds, metabolic programs, and tissue-supportive functions according to the physiological state of the organ. In the heart, sympathetic and parasympathetic signaling influence resident macrophage populations involved in electrical conduction and tissue repair [25]. In the intestine, muscularis macrophages form reciprocal circuits with enteric neurons through CSF-1 and BMP2 signaling, coordinating gut motility and neuronal survival [33, 233]. In the skin, dermal macrophages interact with sensory neurons that regulate their survival, anti-inflammatory programming, and regenerative functions [249, 254]. Together, these observations extend concepts originally established in CNS neuroimmunology to multiple organs, opening avenues for the development of new therapeutic strategies.

## Therapeutic Approaches for TRMS: Strategies and Perspectives

Despite their remarkable tissue-specific specialization, the macrophage populations discussed throughout this review share several common biological principles. Their physiological functions are established through developmental layering and reinforced by niche-derived signals that shape transcriptional, epigenetic, and metabolic identity [267]. During infection, these programs may be disrupted through inflammatory reprogramming, loss of niche signals, depletion of resident cells, or replacement by recruited monocyte-derived macrophages [268]. Although the consequences differ across organs, the underlying process is similar: infection compromises tissue-imprinted macrophage identity. Consequently, many emerging therapeutic strategies converge on a common objective, preserving, restoring, or recreating the niche conditions that sustain resident macrophage function.

Recognition of TRMs as organ-function regulators has shifted therapeutic horizons from broad immunosuppression to precision immunomodulation [269]. The goal is no longer to suppress the immune system but to recalibrate macrophage ecosystems, selectively modulating recruitment, differentiation, and functional polarization within specific tissues (Table 2). These approaches map onto the three physiological axes we have outlined: heart-brain axis interventions: Neuromodulation (vagal stimulation, β2-selective blockade) can reshape cardiac macrophage population [25, 270, 271]. liver-spleen axis targeting: Iron metabolism modulation, hepcidin mimetics, or CSF-1 therapy can restore systemic filtering capacity mediated by liver and splenic macrophages [272, 273]. barrier tissue strategies: Metabolic reprogramming (PPARγ agonists in lung, AHR agonists in gut) can reinforce tolerogenic programs [37, 274].

**Table 2:** Host-directed therapeutic strategies and precision immunomodulation of macrophage dynamics

Strategy	Mechanism	Molecular Target & Cellular Pathway	Target Disease & Clinical Objective	Current Translational Status	Primary macrophage population targeted	References
Metabolic Reprogramming	Metformin	AMPK activation; enhancement of mitochondrial ROS and autophagy.	**Tuberculosis**: Intracellular bacterial clearance. **Chagas Disease**: Attenuation of myocardial inflammation and cardiac dysfunction.	Tb: Phase 2 Clinical Trial (adjunct). CD: Preclinical.	Intracellularly infected tissue macrophages.	[216, 275–278]
Nanoparticle-Mediated Target Delivery	Iron Oxide Nanoparticles (IONPs)	Passive phagocytic uptake; lysosomal dissolution driving Fenton chemistry and hydroxyl radical production.	**Visceral Leishmaniasis**: Direct oxidative destruction of amastigotes and inflammatory polarization.	Preclinical.	Phagolysosomal niches within splenic and hepatic macrophages.	[279]
Adoptive Cell Therapy	CAR-Macrophages (CAR-M)	Genetically engineered chimeric receptors to initiate targeted phagocytosis and antigen presentation.	**MDR Bacteria (MRSA)**: Direct clearance of refractory tissue infections.	Preclinical.	Skin macrophages.	[280]
Autologous Cell Transfer	Macrophage Infusion Therapy	Ex vivo differentiation of patient-derived peripheral blood monocytes into a restorative phenotype.	**Liver Cirrhosis**: Stimulation of extracellular matrix remodeling and suppression of local fibrogenesis.	Phase 2 Completed - MATCH Trial.	Hepatic sinusoidal and stellate cell-adjacent microenvironments.	[281]
Stratified Biomarker-Guided Therapy	Targeted Cytokine/Anti-Cytokine Platforms	Patient stratification via ferritin and HLA-DR expression; allocation to IL-1R blockade or recombinant IFN-γ.	**Sepsis-Induced Immune Dysregulation**: Precision balance between hyper-inflammation and systemic immunoparalysis.	Phase 2 Complete - ImmunoSep.	Systemic splenic, hepatic, and circulating myeloid lineages.	[282]
Trained Immunity	Innate Immunomodulatory Vaccines	Epigenetic reprogramming of myeloid lineages.	**Chagas Disease**: Prophylactic or therapeutic synergy with adaptive T-cell responses for infection control.	Preclinical/Early Clinical Assessment.	Embryonically derived and newly recruited cardiac resident networks.	[283]

### Targeting the Recruitment Axis

In conditions where recruited monocytes drive pathology (e.g., severe ARDS or fulminant myocarditis), inhibiting their entry may preserve the resident niche. Excessive recruitment through chemokine pathways such as CCL2–CCR2 drives tissue damage in the heart, lung, and liver during infection [48, 284, 285]. Pharmacological inhibition of CCR2 or targeted silencing of CCR2 expression using lipid or polymer-based nanoparticles has shown efficacy in preclinical models [286, 287], reducing inflammatory macrophage accumulation while sparing embryonically derived resident populations. Conversely, enhancing the CX3CL1–CX3CR1 pathway may support the survival of resident populations, providing a dual-action approach [288, 289].

Reinforcing resident macrophage identity has emerged as a complementary strategy [290]. Signals such as IL-10 [291], IL-4 [292], IL-33 [293], and CSF-1 [294] support resident macrophage survival and reparative programming across tissue. For example, IL-33–driven activation of tissue-resident macrophages promotes repair in the heart and skeletal muscle, protecting against chronic rejection or infection-related inflammation [48, 293, 295].

### Metabolic Reprogramming (Host-Directed Therapies)

Metabolic reprogramming represents a promising therapeutic lever for modulating macrophage-mediated responses during active infection and subsequent tissue repair. Under steady-state homeostatic conditions, fully differentiated TRMs rely predominantly on oxidative phosphorylation, efficient lipid handling, and high mitochondrial fitness to sustain their long-term survival and execute tissue-specific physiological tasks within their respective niches [296–298]. This oxidative and fatty acid-driven metabolic wiring supports baseline homeostatic and reparative programs, fundamentally distinguishing established resident cells, regardless of whether their origin is embryonic or monocyte-derived, from acutely recruited inflammatory macrophages that shift toward aerobic glycolysis to drive pro-inflammatory loops [299, 300]. Consequently, pharmacological strategies designed to reinforce mitochondrial respiration or regulate lipid utilization have emerged as viable approaches to stabilize protective, tissue-imprinted macrophage phenotypes. Selective activation of PPARγ, which drives the programming of mitochondrial fatty-acid oxidation [301], and targeted manipulation of intracellular iron and heme processing [302], have all demonstrated therapeutic efficacy in reinforcing these homeostatic programs and preserving tissue homeostasis in preclinical models.

Metformin, a common diabetes drug, activates AMPK, forcing macrophages to switch metabolism [275]. In preclinical models of *M. tuberculosis* and *M. avium* infection, metformin restricts intracellular bacillary replication by upregulating ROS production and activating autophagy pathways, thereby enhancing phagolysosomal killing capacity [215, 216, 276]. Transitioning to clinical evaluation, findings from Phase 2 randomized controlled trials demonstrate that while adjunctive metformin added to standard anti-tuberculosis therapy (ATT) does not accelerate sputum culture conversion, it effectively attenuates excessive pathogen-induced inflammation [277, 303]. This immunomodulatory activity minimizes pulmonary parenchymal damage, resulting in accelerated resolution of lung lesions. Beyond tuberculosis, this host-directed metabolic intervention is being evaluated in clinical trials for chronic infections caused by *M. leprae* [304]. Because leprosy reactions represent the primary etiology of irreversible peripheral nerve damage and subsequent physical disability in multibacillary patients, modulating the underlying innate immune response is a critical clinical objective.

Metformin treatment of *T. cruzi* infected mice also leads to reduced cardiac inflammation, improved CD8 + T cell function and a lower parasitemia, likely through the modulation of macrophage activation [278]. Similarly, the immunomodulation of innate cells with vaccination may complement that of the adaptive response and provide improved *T. cruzi* control [278, 283].

By reshaping macrophage metabolism and transcriptional programs, these approaches aim to restore immune balance, enhance pathogen clearance, and limit tissue damage during infection.

### Iron-Targeted Therapies and Nanoparticle Delivery Systems

The iron-handling functions of Kupffer cells and red pulp macrophages described in the liver–spleen section provide the biological rationale for iron-targeted therapeutic strategies: when these populations are overwhelmed by ferroptosis, iron-overloaded, or functionally subverted by intracellular pathogens, their antimicrobial capacity fails [305].

In this sense, iron metabolism has emerged as a critical regulator of macrophage function during infection and inflammation. Dysregulated iron handling can promote oxidative stress and trigger ferroptosis, a form of regulated cell death characterized by iron-dependent lipid peroxidation [306]. During infections, excessive labile iron promotes Fenton chemistry, generating highly reactive hydroxyl radicals that damage membranes, proteins, and DNA, ultimately compromising macrophage viability and antimicrobial capacity [307, 308]. Consequently, iron-targeted therapies have been proposed as strategies to preserve macrophage function and limit ferroptosis-driven tissue injury.

Deferiprone, an orally available iron chelator approved for the treatment of transfusional iron overload, has demonstrated protective effects in models of malaria [309], sepsis [310], and toxoplasmosis [311], where it preserves macrophage viability and reduces pathogen-induced tissue damage. By sequestering labile intracellular iron, deferiprone reduces the substrate available for Fenton reactions, thereby limiting oxidative damage and lipid peroxidation [312].

Parallel to these metabolic interventions, advances in nanotechnology are enabling more precise targeting of macrophages during infection. Because macrophages are professional phagocytes, they naturally internalize nanoparticles through endocytosis and phagocytosis. This intrinsic property allows nanoparticles to function as passive targeting systems, delivering therapeutic molecules directly to macrophage compartments. Iron oxide nanoparticles (IONPs), for example, have been explored as “Trojan horse” systems in host-directed antimicrobial strategies [279].

Together, iron-targeted therapies and nanoparticle-based delivery systems illustrate emerging host-directed approaches to manipulate macrophage biology during infection. By combining metabolic modulation with targeted delivery technologies, these approaches aim to preserve macrophage viability, enhance pathogen clearance, and reprogram detrimental macrophage phenotypes within infected tissues.

### Chimeric Antigen Receptor Macrophages (CAR-M)

Adoptive cell therapy is expanding beyond T lymphocytes to include macrophage-based approaches. Chimeric Antigen Receptor Macrophages (CAR-Ms) are genetically engineered macrophages expressing synthetic receptors designed to recognize specific pathogen-associated or tumor antigens [313]. The ability of macrophages to reside persistently within solid tissue microenvironments, a property central to the biology of tissue-resident macrophages described throughout this review, provides a key biological justification for macrophage-based adoptive cell therapy, since engineered macrophages can potentially be directed to durable tissue niches inaccessible to T cells.

Upon antigen engagement, CAR-Ms become activated and trigger phagocytosis, antigen processing, and inflammatory signaling, enhancing response to therapy for patients with non-responsive tumors [314]. However, it is important to address that current CAR-M platforms derive their cellular substrate from peripheral blood monocytes differentiated ex vivo under defined cytokine conditions. These cells, while macrophage-like in phenotype, do not carry the tissue-specific epigenetic programs of embryonically derived TRMs [315]; future engineering strategies may seek to incorporate niche-identity signals into the differentiation protocol to enhance tissue persistence and reduce inflammatory bystander activation.

In addition to direct phagocytosis, CAR-Ms can modulate the immune microenvironment by secreting cytokines, presenting antigens to T cells, and recruiting other innate immune cells [316, 317]. Recent studies have begun to explore CAR-M platforms against infectious diseases [318]. CAR-Ms targeting the methicillin-resistant *S. aureus* (MRSA) have shown promising results by promoting phagocytosis and intracellular MRSA clearance [280].

Despite these promising advances, several challenges remain before CAR-M therapies can be broadly translated into clinical practice. These include controlling macrophage activation to prevent excessive inflammation, ensuring sufficient persistence of engineered cells in vivo, and overcoming macrophage plasticity that may drive immunosuppressive phenotypes in certain microenvironments [319]. Advances in gene-editing technologies, receptor design, and metabolic programming are expected to address these limitations and further expand the therapeutic potential of CAR-M approaches in infectious and inflammatory diseases [320].

### Clinical Trials

Translating macrophage biology into therapeutic interventions has become an emerging focus of clinical research. One of the most notable examples is the MATCH trial in patients with liver cirrhosis, representing a landmark Phase II study investigating autologous macrophage therapy [281]. In this approach, peripheral blood monocytes were isolated from patients and differentiated ex vivo into restorative macrophages before being reinfused. The trial demonstrated an acceptable safety profile and encouraging exploratory clinical signals, and although the primary MELD-score endpoint was not met; these results support continued investigation of macrophage-based cell therapy [281].

Beyond adoptive cell therapy, macrophage immunobiology is informing targeted, host-directed stratification mechanisms during severe systemic inflammatory syndromes. The international ImmunoSep trial represents a major advance in personalized medicine for sepsis, where patient cohorts are rapidly stratified via localized biomarkers, specifically tracking hyperferritinemia and monocyte HLA-DR expression dynamics [282]. These immunophenotypic signatures allow clinicians to delineate two mutually exclusive, actionable endotypes frequently obscured by syndrome-based labels: macrophage activation-like syndrome (MALS) and sepsis-induced immunoparalysis. As demonstrated in the peer-reviewed trial results [282], patients presenting with MALS-associated hyperinflammation benefit significantly from targeted IL-1 receptor blockade using anakinra. Conversely, individuals exhibiting severe innate immunoparalysis receive recombinant human IFN-γ to safely reverse monocyte dysfunction and restore macrophage antimicrobial competence, establishing a scalable avenue for precision immunotherapy in critical care.

As regulators of organ homeostasis, TRMs integrate immune sensing with tissue-specific functions across multiple physiological axes. Within the heart–brain axis, neuromodulatory signals can shape macrophage-mediated control of inflammation and tissue repair. In the liver–spleen axis, interventions targeting iron metabolism and systemic filtering capacity may restore immune balance during infection and sepsis. At barrier tissues, metabolic and environmental signals orchestrate macrophage programs that maintain tolerance while preserving antimicrobial defense. The expanding therapeutic toolbox, including metabolic reprogramming, iron modulation, nanoparticle-based targeting, CAR-macrophage engineering, and niche-directed cell therapies, demonstrates the feasibility of manipulating these systems with increasing spatial and functional precision. As knowledge of TRM ontogeny, niche signaling, and epigenetic programming continues to evolve, future therapies will likely integrate immunometabolic modulation with tissue-specific targeting and biomarker-guided interventions. In this context, the goal of therapy shifts from dampening inflammation to restoring the macrophage-centered circuits that sustain tissue physiology, opening new avenues for precision immunomedicine in infectious and inflammatory diseases.

## Conclusion

TRMs function as specialized cellular components that integrate immune surveillance with the physiological demands of individual organs. By framing immunity within broader organ systems, such as the neuroimmune heart-brain circuit, the metabolic liver-spleen iron axis, and highly exposed barrier surfaces, this review outlines a conceptual model where infection outcomes depend on the functional stability of the resident macrophage niche. Inflammatory stress and monocyte recruitment can disrupt these tissue-imprinted programs, leading to long-term alterations in organ function that outlast active pathogen clearance. Future research focusing on the precise molecular cues that maintain or restore resident macrophage identity may inform targeted, host-directed therapeutic strategies designed to minimize immunopathology and prevent post-infectious structural sequelae.

## Data Availability

No datasets were generated or analysed during the current study.
